# Multi-Compartment Spatially-Derived Radiomics From Optical Coherence Tomography Predict Anti-VEGF Treatment Durability in Macular Edema Secondary to Retinal Vascular Disease: Preliminary Findings

**DOI:** 10.1109/JTEHM.2021.3096378

**Published:** 2021-07-12

**Authors:** Sudeshna Sil Kar, Duriye Damla Sevgi, Vincent Dong, Sunil K. Srivastava, Anant Madabhushi, Justis P. Ehlers

**Affiliations:** 1 Department of Biomedical EngineeringCase Western Reserve University 2546 ClevelandOH44106USA; 2 The Tony and Leona Campane Center for Excellence in Image-Guided Surgery and Advancing Imaging ResearchCleveland Clinic Cole Eye Institute 372252 ClevelandOH44106USA

**Keywords:** Diabetic macular edema (DME), Intravitreal Aflibercept Injection (IAI), optical coherence tomography (OCT), radiomics, vascular endothelial growth factor (VEGF)

## Abstract

Objective: Diabetic macular edema (DME) and retinal vein occlusion (RVO) are the leading causes of visual impairments across the world. Vascular endothelial growth factor (VEGF) stimulates breakdown of blood-retinal barrier that causes accumulation of fluid within macula. Anti-VEGF therapy is the first-line treatment for both the diseases; however, the degree of response varies for individual patients. The main objective of this work was to identify the (i) texture-based radiomics features within individual fluid and retinal tissue compartments of baseline spectral-domain optical coherence tomography (SD-OCT) images and (ii) the specific spatial compartments that contribute most pertinent features for predicting therapeutic response. Methods: A total of 962 texture-based radiomics features were extracted from each of the fluid and retinal tissue compartments of OCT images, obtained from the PERMEATE study. Top-performing features selected from the consensus of different feature selection methods were evaluated in conjunction with four different machine learning classifiers: Linear Discriminant Analysis (LDA), Quadratic Discriminant Analysis (QDA), Random Forest (RF), and Support Vector Machine (SVM) in a cross-validated approach to distinguish eyes tolerating extended interval dosing (non-rebounders) and those requiring more frequent dosing (rebounders). Results: Combination of fluid and retinal tissue features yielded a cross-validated area under receiver operating characteristic curve (AUC) of 0.78±0.08 in distinguishing rebounders from non-rebounders. Conclusions: This study revealed that the texture-based radiomics features pertaining to IRF subcompartment were most discriminating between rebounders and non-rebounders to anti-VEGF therapy. Clinical Impact: With further validation, OCT-based imaging biomarkers could be used for treatment management of DME patients.

## I. Introduction

Diabetic retinopathy (DR) is a progressive, chronic microvascular disorder that may be accompanied with vision-threatening complications, such as diabetic macular edema (DME). The excessive retinal vascular permeability caused by the damage to the blood-retinal barrier leads to retinal fluid accumulation within the macular region [1], [2]. Retinal vein occlusion (RVO) occurs typically due to intravascular thrombosis and may also result in macular edema secondary to increased vascular permeability. Vascular endothelial growth factor (VEGF) has been recognized as a critical cytokine that induces retinal vascular hyperpermeability in both DME and RVO [3] by stimulating the breakdown of the intercellular junction of the blood-retinal barrier. This in turn causes the accumulation of intraretinal fluid (IRF) and/or subretinal fluid (SRF) within the retina. Elevated VEGF levels in eyes with RVO and DME may also exacerbate microangiopathy and ischemia [4].

Multiple studies have explored the effectiveness of anti-VEGF therapy in improving vision and reducing macular edema in both DME and RVO, and it has become the gold-standard first-line therapy for both conditions [5]–[9]. Aflibercept is an FDA-approved VEGF inhibitor that acts as a decoy receptor, binding soluble VEGF and prevents VEGF from binding to retinal cell receptors [10]. Intravitreal aflibercept injection (IAI) results in substantially improved visual acuity and anatomic outcomes for many patients with DME and RVO [11]–[14]. However, there are significant variations in visual outcomes and tolerance of treatment interval extension. Quantification and feature analysis of different compartments of the retina is of the utmost importance for understanding the heterogeneous response to anti-VEGF therapy [15]– [18]. Classification of the response to anti-VEGF in different categories may provide a better understanding of the visual potential of a particular treatment plan [4].

The revolution in ophthalmologic imaging techniques has led to significant advancement in the assessment of clinically silent abnormalities and their objective measurement. It also results in a more precise diagnosis and understanding of disease progression [19]. For the last two decades, spectral-domain optical coherence tomography (SD-OCT) has been shown to have a great impact on clinical management of ocular diseases through improved monitoring of patients, earlier identification of pathology, and more-precise treatment protocols [20]. It is a non-invasive and non-contact imaging method with diverse clinical uses in ophthalmology [21]. OCT images are largely used by ophthalmologists to determine the severity level of DME through a quantitative assessment of retinal thickening and the subjective interpretation of fluid presence [22]. However, clinical interpretation and predictive value of these quantitative features is subject to variability based on the impression of an individual ophthalmologist. Currently, there are no well-established, non-invasive imaging biomarkers to predict response to anti-VEGF therapy. Developing predictive biomarkers is, therefore, of high significance for the therapeutic decision-making and better assessment of response to anti-VEGF treatment.

Radiomics refers to the extraction and analysis of extensive advanced quantitative imaging features from medical images using computer vision and image processing techniques [23]. In many cases, the goal of the radiomics-based analysis is to identify the association between biologically relevant features extracted from the clinical images to support clinical decision-making [24]. Previous studies have demonstrated the efficacy of textural-based radiomics features in predicting therapeutic response from the ultra-widefield fluorescein angiography (UWFA) images [25], [26], investigating the role of FA-derived leakage morphology and vessel tortuosity-based biomarkers to discriminate eyes that required more frequent dosing from those who did not. However, to the best of our knowledge, the implications of the OCT-derived morphologic biomarkers in assessing the anti-VEGF treatment response have not been rigorously investigated.

Additionally, previous works on radiomics [27], [28] in oncology have evaluated the role of texture-based radiomics features from different tumor subcompartments for predicting treatment response across a range of cancer types. This therefore begs the question whether radiomic features extracted from specific compartments in the eye as identified on OCT scans (e.g., various fluid compartments and/or tissue compartments) are with likelihood of treatment response/durability. Remarkable variations in both morphological characteristics as well as texture are observed across the fluid and the retinal tissue compartments in the OCT scans. We hypothesized that the heterogeneity within the different retinal compartments may be well captured by different texture-based radiomic descriptors, and these features will have biologic relevance of underlying sensitivity to anti-VEGF therapy. A key feature of this analysis is the long-term predictive potential of this assessment in that baseline images are assessed for the overall treatment durability that is identified more than six months after this initial assessment.

Consequently, in this preliminary study, we sought to evaluate the degree to which the radiomic features within the individual fluid and the retinal tissue compartments are associated with anti-VEGF treatment response and to identify the specific spatial compartments that contribute the most pertinent features for predicting therapeutic response. Radiomic features were extracted from each of the fluid compartment (IRF and SRF) and various retinal tissue compartments [i.e., Internal Limiting Membrane (ILM) to Retinal Pigment Epithelium (RPE), ILM to Ellipsoid Zone (EZ), EZ to RPE] on SD-OCT scans obtained from the PERMEATE [1] clinical trial, a prospective open-label IRB-approved study investigating potential imaging biomarkers in eyes with DME and RVO undergoing treatment IAI.

The organization of the rest of this paper is as follows. A review of the previous works and novel contributions of this study are presented in Section II. Section III describes the spatial compartmental approach for assessment of the predictive relevance of radiomic features from the OCT scans. Experimental results are presented in Section IV, with a discussion of the findings in Section V. Concluding remarks are presented in Section VI.

## II. Previous Related Work and Novel Contributions

Extensive previous research has demonstrated the utility of radiomics analysis for diagnosis, prognosis, and treatment of several diseases, including brain tumors [29], breast cancer [30], lung cancer [31], [32], prostate cancer [33], and rectal cancer [34]. The majority of previous work in computational image analysis of OCT scans for DME and DR has focused on automated multi-layer retinal segmentation, fluid feature extraction, and targeted *en face* and zonal mapping applications [15]–[18], [20], [21]. While there is an unmet need of developing new computationally-derived imaging biomarkers of outcome and anti-VEGF treatment response to improve clinical management of the patients treated with IAIs, the role of radiomic features in predicting treatment response for ocular diseases has not been extensively investigated. In a recent study [25], Prasanna *et al.* explored two novel UWFA-derived radiomics biomarkers, leakage distribution and vessel tortuosity to predict therapeutic durability of IAIs. The first biomarker identified captures the discrepancies that exist in the spatial arrangement of leakage patterns between the eyes that more likely tolerate extended interval dosing as compared to those that do not. The second one was related to the vessel tortuosity, which identifies greater disorder and more complex tortuosity patterns in retinal vasculature for the eyes that had decreased tolerance to extended interval dosing. In another attempt to distinguish the favorable response of anti-VEGF therapy based on the morphological and tortuosity features extracted from the baseline UWFA images, Moosavi *et al.* identified that the proximity of leakage foci to the vessels have higher variance in eyes who have more durable treatment response [26]. Also, the local tortuosity of the vessels in the vicinity of the leakage foci was found to be higher for the patients that do not tolerate extended interval dosing.

The Phase III clinical trials established IAI as first-line therapy in the management of macular edema retinal vascular disease, showing an overall improvement in functional (i.e., visual acuity) and anatomic (i.e., retinal thickness) outcomes [12]–[14]. However, the optimal personalized approach to predict which eyes will tolerate extended interval dosing compared to high-frequency treatment (e.g., monthly) remains elusive. In an effort to explore imaging biomarkers, studies [18] have investigated retinal fluid features and EZ integrity dynamics on SD-OCT in eyes with DME treated with IAIs. Retinal fluid metrics have been analyzed quantitatively and a novel OCT biomarker, the “retinal fluid index (RFI)”, was introduced. Ehlers *et al.* demonstrated that the volatility of RFI was associated with intolerance to the treatment interval extension in eyes treated with IAI for DME [16]. Given that OCT provides a very good visualization of the intraretinal and subretinal cystoid fluid, a fast-automated quantification of retinal fluid on OCT images based on supervised learning was presented in [15].

The different fluid and retinal tissue compartments of the OCT images contain valuable information in the form of variation of texture, gradient, and heterogeneity. Subtle variation in morphological characteristics and alternation in texture within the fluid and retinal tissue compartments as a result of the underlying disease manifestation are well captured by SD-OCT.

Recently deep learning (DL)-based models have been evaluated for assessment of various ocular diseases, including diabetic eye disease [35]–[38]. Rasti *et al.* utilized a novel deep convolutional neural network (CNN) using pre-treatment OCT scans as the input for predicting differential retinal thickness following three consecutive anti-VEGF injections with 5-fold cross-validation [35]. The attention-based CNN model presented in [35] preserves and highlights the global structures in OCT images and enhances local features from fluid/exudate-affected regions to efficiently use retinal thickness information for response prediction. Beyond DME and DR, an additional study evaluated a CNN-based model’s capacity for predicting effectiveness of anti-VEGF therapy for choroidal neovascularization (CNV) in 228 patients [36] using a modified ResNet-50 model. Different DL models such as AlexNet, GoogleNet, VGG-16, ResNet-50 were trained separately using OCT images and were employed to segment lesion regions. The ResNet-50 model achieved an AUC of 0.91 to predict the effect of anti-VEGF therapy for CNV response and demonstrated that the full OCT images performed better than the lesion-specific regions. Utilizing a dataset that included 183,402 OCT B-scans, Prahs *et al.* [37] developed a DL algorithm to distinguish retinal OCT B-scans that require an intravitreal injection from those that do not require an injection, achieving 95.5% accuracy on validation dataset. Importantly, this approach did not evaluate treatment response but rather treatment need (e.g., abnormal vs normal). Utilizing a combination of clinical feature variables and OCT image features from 304 eyes with 6,348 clinical variables, Liu *et al.* [38] developed an ensemble machine learning system consisting of four DL models and five classical machine learning models to predict the post treatment central foveal thickness (CFT) and best corrected visual acuity (BCVA) after the initial 3 monthly anti-VEGF injections.

These previous reports show promising results for the potential for DL methods in larger datasets in the identification of image features that predict the need for treatment and for predicting initial treatment response. However, the studies have not extensively evaluated specific retinal subcompartments and have also not evaluated treatment interval tolerance following initial treatment induction. The ability to identify and predict which patients will respond to treatment extension and long-term therapy is a critical component for personalized therapeutic decision-making. The identification of the spatial subcompartments that contribute the most pertinent features to discriminate between the rebounders and the non-rebounders following treatment extension remain unexplored in the existing literature. The novel contribution of the work in this report is to evaluate the ability of texture-based radiomics features pertaining to individual OCT subcompartments to characterize the response patterns of eyes treated with anti-VEGF therapy, and to identify the specific fluid and/or retinal tissue compartments that contribute the most pertinent features for predicting therapeutic response. In addition, this radiomics-based assessment of imaging features demonstrates the potential critical value of this technology in smaller datasets where DL alone may not be optimal.

In addition to radiomics analysis, a DL model is used to identify the key contributing regions that distinguish the rebounders and the non-rebounders. The rationale for using DL-based approach is to identify the key subcompartments that contain the most discriminating features and to establish the relevance and importance of the spatial compartment-based analysis characterized by the radiomics features. The DL-based generated visual class activation map (CAM) helps validate the importance of the regions from where the engineered radiomic features were extracted.

**FIGURE 1.**
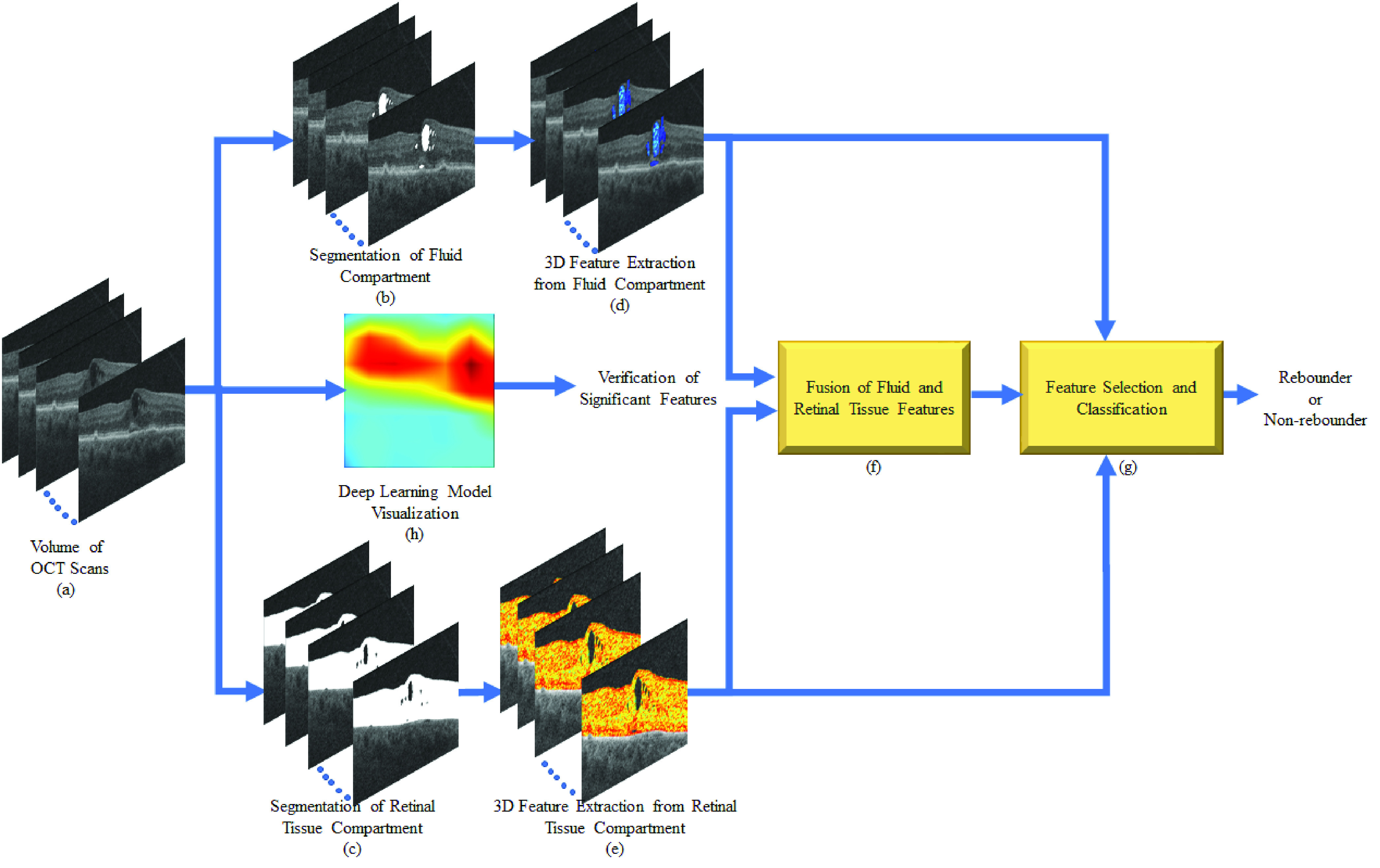
*Workflow of the OCT-derived spatial radiomic features from the PERMEATE study and corresponding evaluation for each patient:* (a) Original OCT scans, Segmentation of (b) Fluid and (c) Retinal tissue compartment, feature extraction from (d) Fluid and (e) Retinal tissue compartments, (f) Fusion of fluid and retinal tissue features, (g) Feature selection and classification, (h) Deep learning based visual CAM to identify the region that contains the most distinctive features.

The schematic diagram of the radiomics-based feature extraction from the baseline OCT images obtained from the PERMEATE study and their evaluation is illustrated in Fig. 1. In the present study, baseline OCT scans (Fig. 1(a)) were segmented utilizing an automated machine-learning augmented segmentation platform with manual correction as needed, (Fig. 1(b) and 1(c), respectively) as previously described [18], [39]. This was followed by radiomics feature extraction from the different fluid and the retinal tissue compartments (Fig. 1(d) and 1(e)). The fluid and the retinal tissue features were fused (Fig. 1(f)) and the top-performing features were selected and fed to the machine learning classifier to distinguish the treatment response between the rebounders and the non-rebounders (Fig. 1(g)). In addition, a DL-based visual attention map was also generated (Fig. 1(h)) to corroborate the regions identified by radiomics-based features that have high prediction power. The detailed description of the individual steps is presented below.

**FIGURE 2.**
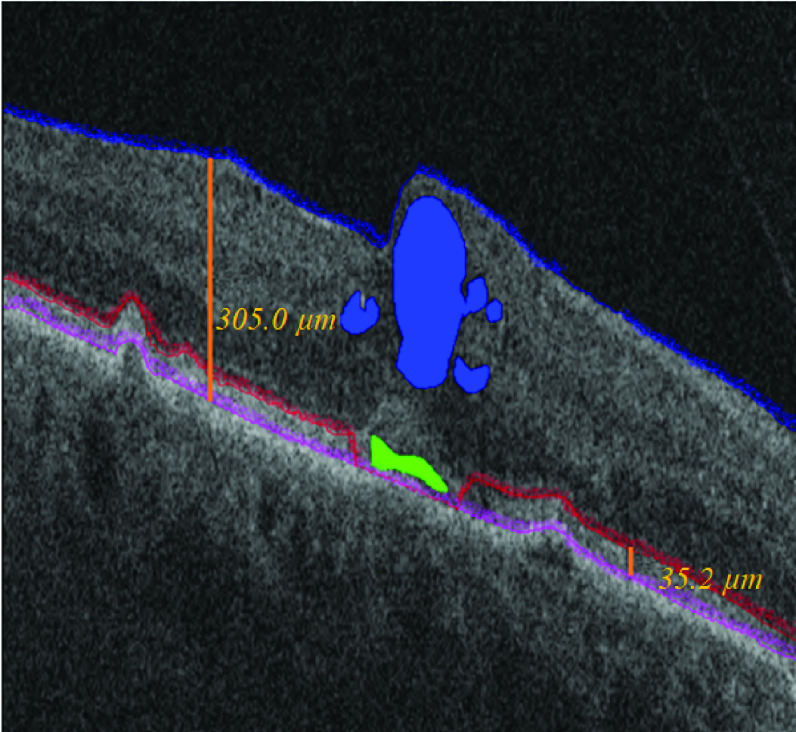
Semi-automated segmentation of foveal B-scan demonstrates volumetric IRF and SRF segmentations in blue and green objects respectively. Retinal layers were segmented for ILM, EZ and RPE layers using blue, red and pink lines respectively. Retinal tissue compartment is defined as the volume between ILM and RPE. The thickness of the retinal compartment is represented by the orange line on the right in this scan ($305.0~\mu \text{m}$). Subretinal compartment is defined as the volume between EZ and RPE. The thickness of the subretinal compartment is demonstrated by the orange line on the left ($35.2~\mu \text{m}$). EZ line segmentation was dropped to RPE line in the presence of subretinal fluid. IRF, intraretinal fluid; SRF, subretinal fluid; ILM, inner limiting membrane; EZ, ellipsoid zone; RPE, retinal pigment epithelium.

## III. Materials and Methods

### A. Dataset Description

The PERMEATE study is an IRB-approved included 28 eyes of 31 patients all of whom provided written informed consent for participation in the study; three participants were excluded due to poor image quality, insufficient follow-up, or patient drop-out during the study. Patients of 18 years or older with foveal-involving retinal edema secondary to DME or RVO based on SD-OCT and Early Treatment Diabetic Retinopathy Study (ETDRS)-based BCVA of 20/25 or worse were qualified to include in the study [11]. IAI injections were applied to the patients in two phases over a period of 12 months. Patients were given two milligrams of IAI monthly [i.e., every four weeks (q4 doses)] for the first six months followed by bimonthly dosing [i.e., for every eight weeks (q8 dosing)] starting from month six. This interval extension was the therapeutic challenge for individual tolerance treatment extension and the specific durability of IAI. Patients were evaluated monthly with SD-OCT. Key endpoints of the PERMEATE study, including mean change in ETDRS-based BCVA, mean change in total leakage index, change in mean central subfield thickness, and mean change in the ischemic index, were assessed. Eyes were categorized into two groups based on the treatment response to the first eight-week therapeutic challenge: rebounders (N = 11) and non-rebounders (N = 17).

The dataset contains a volume of 128 baseline OCT scans for each patient. The central 43–85 slices were utilized for radiomics-based assessment of the central macular area. The empty slices within the volume were excluded and the remaining slices were used for extracting fluid features for each patient. The mid central subfield slices (Slice 43–85) were used for feature extraction from retinal tissue compartments. The rebounders showed loss of BCVA and frequently associated increased macular edema during the first eight-week challenge, whereas the non-rebounders maintained or improved in BCVA [26].

### B. Region of Interest/Image Segment

Macular cube scans with $512\times 128$ A-scans covering a nominal $6\times 6$ millimeter scan area was imported to a machine learning enhanced automated retinal layer segmentation and feature extraction platform, OCTViewer (Cleveland Clinic, Cleveland, Ohio) as previously described [18], [39]. The tool performed automated segmentation of ILM, EZ, RPE bands, and IRF and SRF objects. The software-generated segmentations were then manually reviewed for all the 128 slices of each scan by trained readers who went through a standardized training for OCT scan segmentation of higher order features. The feature extraction platform enabled manual corrections of segmentation errors by the readers when required. The segmentation line for ILM was placed on top of the retina at the vitreoretinal junction. EZ and RPE segmentation lines were placed in the middle of the corresponding bands.

Hyporeflective space that increased the distance between EZ and RPE bands were segmented as SRF. Hyporeflective areas that contributed to retinal thickness between ILM and EZ were segmented as IRF. To minimize variability, the image analysis environment was standardized for monitor settings, illumination, and computer configuration. After all of the scans were reviewed and corrected by a trained reader, a senior project lead reviewed all scans and segmentation for segmentation accuracy and consistency between scans.

### C. Spatial Localization of Fluid and Retinal Tissue Compartments

We define an image $I$ as a three-dimensional (3D) spatial grid of voxels corresponding to the volume of OCT scans. Let $I_{F}$ and $I_{RTC}$ represent a sub-volume of $I$ corresponding to the segmentation of fluid and the retinal tissue compartment between ILM and RPE, respectively. From $I_{F}$ we further define intraretinal fluid ($I_{F}^{IRF}$) and subretinal fluid ($I_{F}^{SRF}$) subcompartment volumes. Also, from $I_{RTC}$ we define: ILM to EZ ($I_{RTC1}$) and EZ to RPE ($I_{RTC2}$) subcompartment volumes.

### D. Radiomics Feature Extraction

A variety of three-dimensional (3D) texture-based radiomics features were extracted from the $I_{F}$ and $I_{RTC}$ sub-volumes at on a MATLAB platform (version 2015b; Mathworks, Natick, Mass). We considered 3D feature extraction from the volume of OCT scans for each patient, since extracting 3D features from the $I_{F}$ and $I_{RTC}\mathrm { }$ sub-volumes would provide better quantitative characterization of the heterogeneity than corresponding two dimensional (2D) features from each of the individual 128 OCT slices separately [18], [39].

#### 1) Feature Extraction From $I_{F}$ Sub-Volume

For every voxel within $S_{1}\in \left \{{ I_{F}^{IRF},I_{F}^{SRF} }\right \}$, a total of 962 texture-based radiomics features were extracted. These features included 52 Haralick, 501 Laws energy, 383 Gabor, and 26 co-occurrence of local anisotropic gradient orientation (CoLlAGe) [40] features on a per-voxel basis. The features extracted from $I_{F}^{IRF}$ and $I_{F}^{SRF}$ ($F_{IRF}$ and $F_{SRF}$, respectively) were then combined to obtain the set of combined fluid features ($F_{f}$).

#### 2) Feature Extraction From $I_{RTC}$ Sub-Volume

Similarly, 962 texture-based radiomics features were extracted from every voxel within $S_{2}\in \left \{{ I_{RTC1},I_{RTC2} }\right \}$. The features obtained from $I_{RTC1}$ and $I_{RTC2}$ ($F_{RTC1}$ and $F_{RTC2}$, respectively) were then fused to encapsulate all the retinal tissue features ($F_{RTC}$) from ILM to RPE.

#### 3) Combination of $F_{f}$ and $F_{RTC}$

The $F_{f}$ and $F_{RTC}$ were finally fused to have an insight into the integrated response of the entire OCT features ($F_{OCT}$).

First-order statistics (median, variance, skewness, and kurtosis) from the feature responses of all the voxels within the region of interest were then computed. All feature values were normalized with a mean of zero and a standard deviation of one. The detailed description of the features extracted are presented in Supplementary Material Section I.

### E. Statistical Evaluation

The features $F_{f},F_{RTC}$ and $F_{OCT}$ were fed to machine learning classifiers separately in a supervised way to determine the features that best distinguish the responses between the rebounders and the non-rebounders. The dataset was split into training and test sets with a ratio of 80:20 [26]. We developed our radiomic model on the training set and validated on the test set. For feature and classifier selection, 1000 iterations of three-fold cross-validation within the training set were used. At each iteration of three-fold cross-validation, three feature selection methods including t-test, Wilcoxon-rank-sum, and minimum Redundancy maximum Relevance (mRmR) were used, each of which involved selecting the top 15 features. Finally, the two-best performing features were obtained from the intersection of these top 15 features (selected by each of the feature selection methods). The selection of the most discriminating two features from the consensus (intersection) of different feature selection methods, in turn, reduced the dependency on any single strategy employed for feature selection. The top two features in each fold and run were then used to train four different classifiers: Linear Discriminant Analysis (LDA), Quadratic Discriminant Analysis (QDA), Random Forest (RF), and Support Vector Machine (SVM) in a cross-validation setting. The best performing classifier was selected based on the Area Under Receiver Operating Characteristic Curve (AUC) value. For each classifier, different performance metrics such as Accuracy (ACC), Sensitivity (true positive rate), and Specificity (true negative rate) values were calculated. In addition to supervised classification, an unsupervised hierarchical clustering was also used to assess the features in discriminating the two classes of patients through clustergram analysis [41].

### F. Visual Class Activation Map

A DL-based visual class activation map (CAM) was generated to identify the regions that were seemed to be most relevant for identifying the radiomic signal in order to predict therapeutic response between the rebounders and the non-rebounders. We sought to evaluate whether the most distinctive compartments identified by the supervised approach corroborated to the principal regions identified by the DL strategy. The DL model was implemented to validate the relevance of individual retinal subcompartments from the perspective of response to therapy.

The Gradient-weighted Class Activation Mapping (Grad-CAM) technique, as described in [42], was used to create the CAM in Keras. The neurons in the last convolutional layers of a CNN look for the semantic class-specific information in the image. The gradient information flowing into the last convolutional layer of the CNN was used by Grad-CAM. First, the gradient of the score for a particular class was computed with respect to feature map activations of a convolutional layer. These gradients flowing back were then global average pooled to obtain the neuron importance weights. Next, a weighted combination of activation maps followed by ReLU was executed to take into account the features that have a positive influence on the class of interest, assuming that the negative pixels likely belong to other categories in the image. The CAM was generated in the form of a course heatmap of the class activations over the input images. It represents a 2D grid of scores corresponding to a particular target (output) class. For every location of the input image, the scores represent the attention paid by the network to that particular location with respect to the output class. The color coding scheme of the heatmap identifies regions of importance for the network to perform the object identification task.

In the present work, the dataset was split into training $(S_{tr})$ and test sets ${(S}_{t})$ with a ratio of 80:20. A neural network with ResNet-50 architecture was implemented in Keras. Training and evaluation were performed using each of the slices. Training was done with 50 epochs per $S_{tr}$ and a learning rate of 0.0001.

### G. Targeted Clinical Implication Assessment

For targeted classification efficiency, we evaluated a threshold predictor to direct the overall classifier towards increased accuracy of identification of a specific group of interest (e.g., rebounders). This approach effectively increases sensitivity for identifying one of these groups while decreasing specificity, which could optimize confidence in treatment decision-making for a given group of primary interest.

## IV. Experimental Results

### A. Study Groups

The PERMEATE study [11], [43] identified 17 subjects to be non-rebounders following the eight-week challenge, while 11 subjects were classified as rebounders following the first eight-week challenge. The “rebounder” terminology refers to the phenomenon of clinical worsening, specifically defined by worsening BCVA, but also associated with increased macular edema, following the extension of therapy from four-week intervals to eight-week intervals.

### B. Experiment 1: Distinguishing Eyes Based on Features Extracted From Fluid Compartments

#### 1) Supervised Classification

Of the classifiers considered (Table 1), the LDA classifier resulted in the highest accuracy and AUC in distinguishing the rebounders and the non-rebounders. The LDA classifier discriminated against the favorable rebounders from the non-rebounders with an ACC of 0.71 ± 0.06 from the $F_{f}$ feature pool.

**TABLE 1:** Classification Results of Different Classifiers on $F_{f}$

Features	Classifier	AUC	ACC	Specificity	Sensitivity
$F_{IRF}$	LDA	0.59±0.09	0.67±0.03	0.66±0.03	0.73±0.02
	QDA	0.58±0.06	0.66±0.06	0.58±0.03	0.72±0.04
	RF	0.58±0.04	0.64±0.07	0.58±0.06	0.63±0.04
	SVM (Gaussian Radial basis Kernel)	0.57±0.03	0.65±0.02	0.54±0.01	0.61±0.04
$F_{f}\,\,(F_{IRF}+F_{SRF})$	LDA	0.68±0.09	0.71±0.06	0.72±0.05	0.76±0.03
	QDA	0.64±0.09	0.69±0.06	0.68±0.06	0.70±0.11
	RF	0.63±0.11	0.67±0.08	0.69±0.03	0.66±0.07
	SVM (Gaussian Radial basis Kernel)	0.61±0.02	0.63±0.03	0.66±0.04	0.61±0.06

**FIGURE 3.**
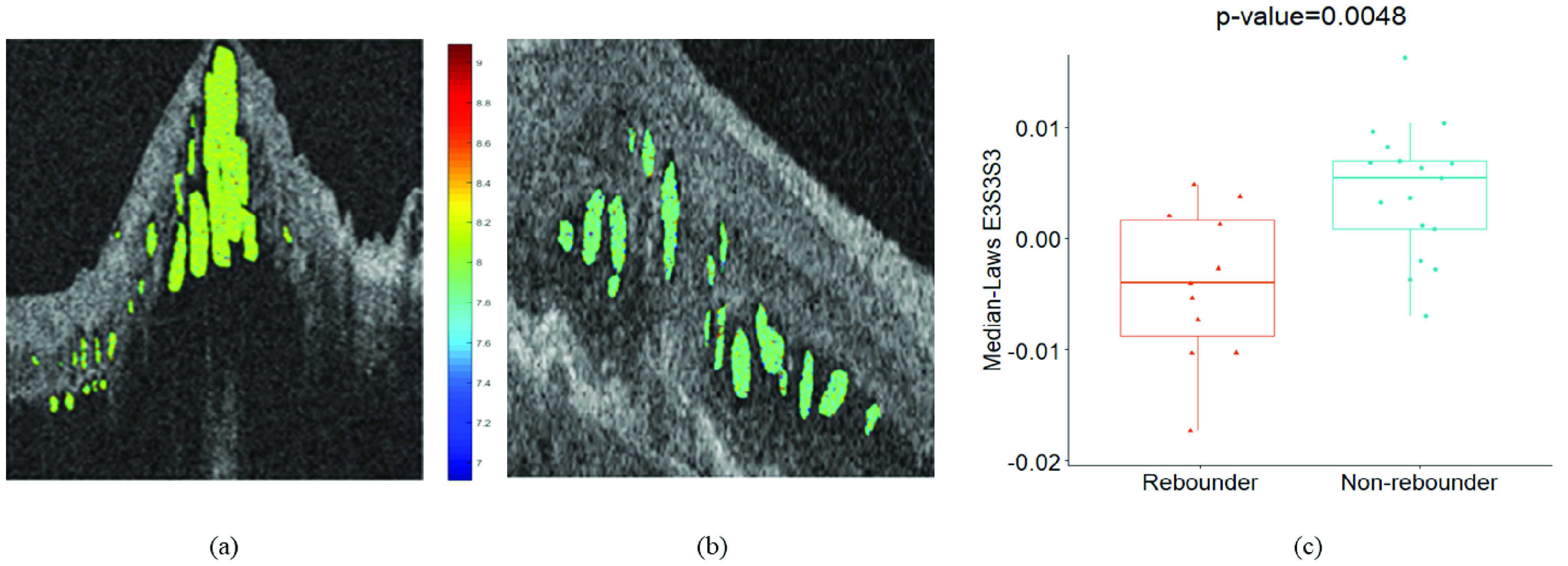
*Feature analysis for fluid compartment:* (a), (b) Feature map of laws E3S3S3 feature for one case of rebounder and non-rebounder, respectively. (c) The box and whisker plot on the left corresponds to the median-laws E3S3S3 feature values from the rebounders (N = 11) and that on the right corresponds to the median-laws E3S3S3 values from the non-rebounders (N = 17).

Median-Laws E3S3S3 IRF feature (p value = 0.0048) was identified as the most discriminating feature during the supervised classification of $F_{f}$. The 3D laws energy kernel E3S3S3 captures the textural patterns of edges (or E) in the horizontal direction, spots (or S) in both the vertical and the diagonal direction using a $3\times 3 \times 3$ convolution filter. The feature map for the Laws E3S3S3 feature is shown in Fig. 3(a) and 3(b), for one case of rebounder and non-rebounder, respectively. The color-coding scheme competently captures the differences between the rebounders and the non-rebounders with a higher expression of the Laws energy descriptor (E3S3S3) evident for the rebounders. The box and whisker plot of the top performing feature is presented in Fig. 3(c). The selection of the Laws E3S3S3 IRF textural feature as the topmost feature ensures that there exist significant differences in heterogeneity within the IRF subcompartments between the rebounders and the non-rebounders.

#### 2) Unsupervised Clustering Analysis

**FIGURE 4.**
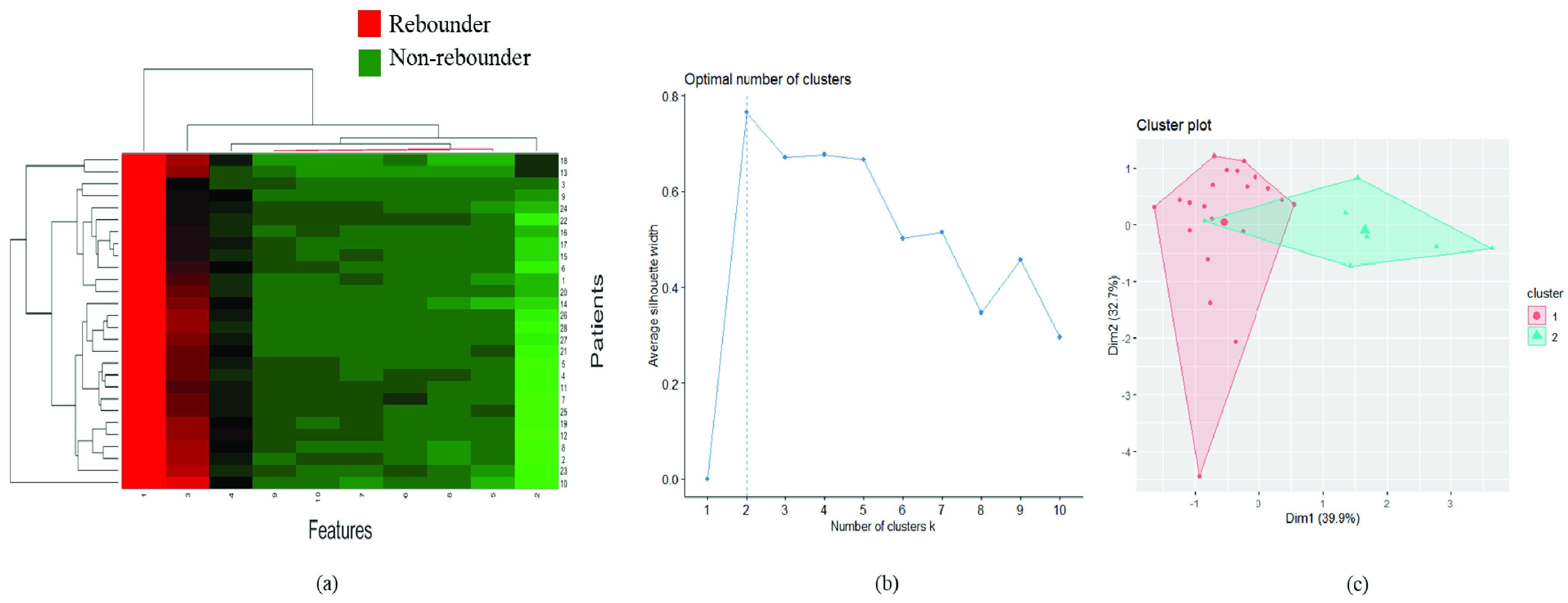
*Unsupervised clustering analysis on fluid features:* (a) The clustergram of radiomics features extracted from the entire fluid compartments. The X-axis represents the reduced-dimension features (10 features selected using PCA) and the Y-axis represents the number of patients. (b) Elbow curve representing an optimum number of clusters formed using the top two principal components after performing PCA on the entire fluid feature set for K-mean clustering analysis. The optimum number of clusters (k) were observed to be two. (c) Clusters after performing clustering using k = 2. Rebounders and non-rebounders are included in cluster 2 and 1, respectively.

Hierarchical clustering [41] is an unsupervised clustering technique used to assess the strength of the identified features in distinguishing the patients into different classes through clustergram analysis. In addition to supervised classification, we also analyzed the patterns associated with the radiomics features extracted from the fluid compartments (IRF+SRF) and assessed their prediction capability using hierarchical clustering [41]. The dimension of the entire $F_{f}$ feature pool was first reduced by Principal Component Analysis (PCA) and the top 10 features were selected followed by unsupervised hierarchical clustering over the reduced dimension, as shown in Fig. 4(a). The rebounder and the non-rebounder classes were represented by red and green clusters, respectively. The red clusters included 49 percent of the rebounders and the green clusters included 62 percent of the non-rebounders.

To measure the efficacy of the radiomics-based features in distinguishing two groups, we also performed K-means clustering. The top two principal components after performing PCA on the entire $F_{f}$ feature set was used for K-means clustering analysis. The optimum number of clusters were obtained by plotting the elbow curve (Figure 4(b)), which was found to be two. 54 percent rebounders and 64 percent non-rebounders were included in cluster 2 and 1, respectively.

### C. Experiment 2: Distinguishing Eyes Based on Features Extracted From Retinal Tissue Compartments

#### 1) Supervised Classification

In the second experiment, we interrogated the entire retinal tissue compartment within the total retinal area (ILM to RPE). Features were extracted from two subcompartments (a) ILM to EZ and (b) EZ to RPE. The fluid feature compartments were subtracted and not included in the retinal tissue assessment. Similar to Experiment 1, the $I_{RTC1}$ and $I_{RTC2}$ features were classified separately using different machine learning classifiers. Finally, the features were combined and classified to evaluate the entire $F_{RTC}$ feature set between ILM to RPE. The different performance metrics corresponding to different classifiers are reported in Table 2. The LDA classifier produced the best result over the other classifiers in terms of AUC and ACC. The ACC values were 0.70 ± 0.02 and 0.65 ± 0.04 for classifying the $F_{RTC1}$ and $F_{RTC2}$, respectively. The classification ACC was 0.75 ± 0.07 for $F_{RTC}$.

**TABLE 2:** Classification Results of Different Classifiers on $F_{RTC}$

Features	Classifier	AUC	ACC	Specificity	Sensitivity
$F_{RTC1}$	LDA	0.65±0.02	0.70±0.02	0.70±0.14	0.74±0.04
	QDA	0.58±0.10	0.65±0.08	0.65±0.21	0.65±0.22
	RF	0.57±0.09	0.63±0.08	0.56±0.26	0.72±0.27
	SVM (Gaussian Radial basis Kernel)	0.54±0.12	0.60±0.06	0.58±0.01	0.62±0.06
$F_{RTC2}$	LDA	0.56±0.04	0.65±0.04	0.71±0.04	0.60±0.03
	QDA	0.53±0.05	0.64±0.02	0.70±0.22	0.55±0.01
	RF	0.54±0.10	0.63±0.08	0.65±0.24	0.56±0.26
	SVM (Gaussian Radial basis Kernel)	0.52±0.02	0.60±0.11	0.62±0.11	0.51±0.04
$F_{RTC}$ ($F_{RTC1}+F_{RTC2}$)	LDA	0.73±0.09	0.75±0.07	0.73±0.06	0.72±0.06
	QDA	0.65±0.09	0.70±0.07	0.70±0.18	0.68±0.19
	RF	0.68±0.09	0.73±0.08	0.76±0.16	0.60±0.20
	SVM (Gaussian Radial basis Kernel)	0.66±0.05	0.73±0.01	0.71±0.04	0.63±0.03

**FIGURE 5.**
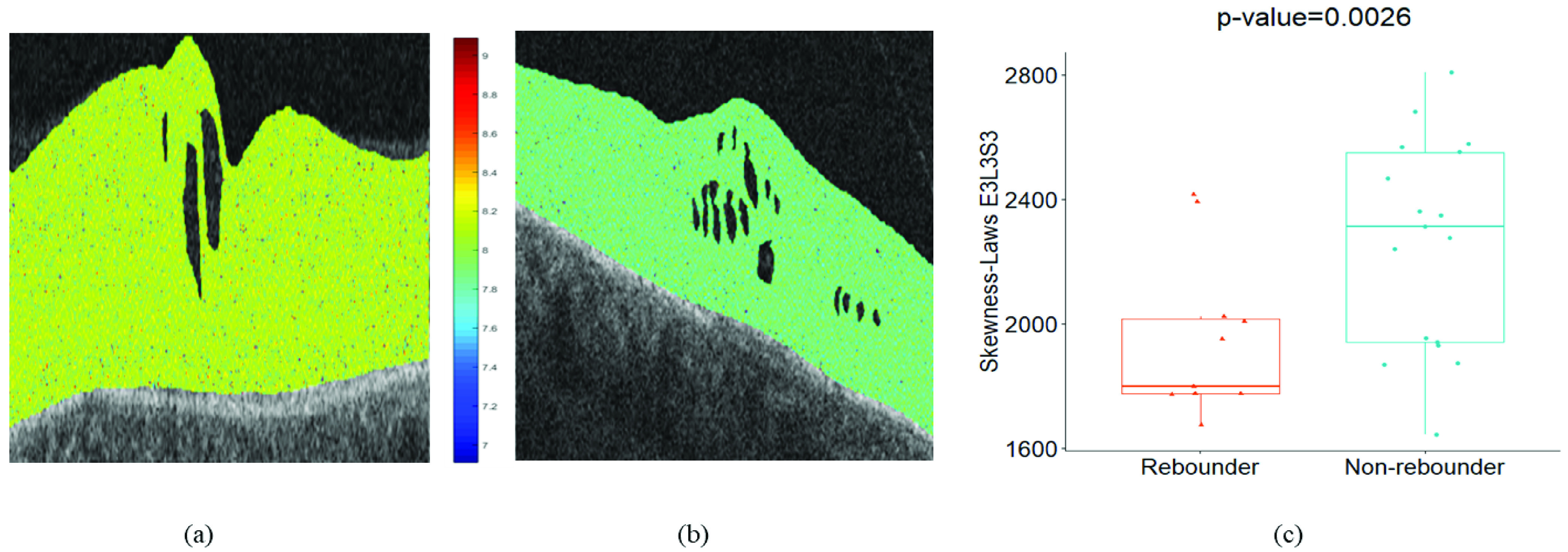
*Feature analysis for retinal tissue compartment:* (a), (b) Feature map of laws E3L3S3 feature for one case of rebounder and non-rebounder. (c). The box and whisker plot on the left corresponds to the skewness-laws E3L3S3 feature values from the rebounders (N = 11) and that on the right corresponds to the skewness-laws E3L3S3 feature values from the non-rebounders (N = 17).

The most discriminating feature identified during classification was Skewness-Laws E3L3S3 (from ILM to EZ) with a p-value = 0.0026. The 3D laws energy kernel E3L3S3 captures the Laws energy-based textural patterns of edges (or E) in the horizontal direction, levels (or L) in the vertical direction and spots (or S) in the diagonal direction using $3\times 3 \times 3$ convolutional kernel. The colors in Fig. 5(a) and 5(b) reflect the significant differences in the feature expression between the rebounder and the non-rebounders, respectively. Thus, the heterogeneity captured within the ILM to EZ appears to play an important role in distinguishing patients into the rebounder and the non-rebounder categories. The box and whisker plot of the feature Skewness-Laws E3L3S3 are presented in Fig. 5(c).

#### 2) Unsupervised Clustering Analysis

**FIGURE 6.**
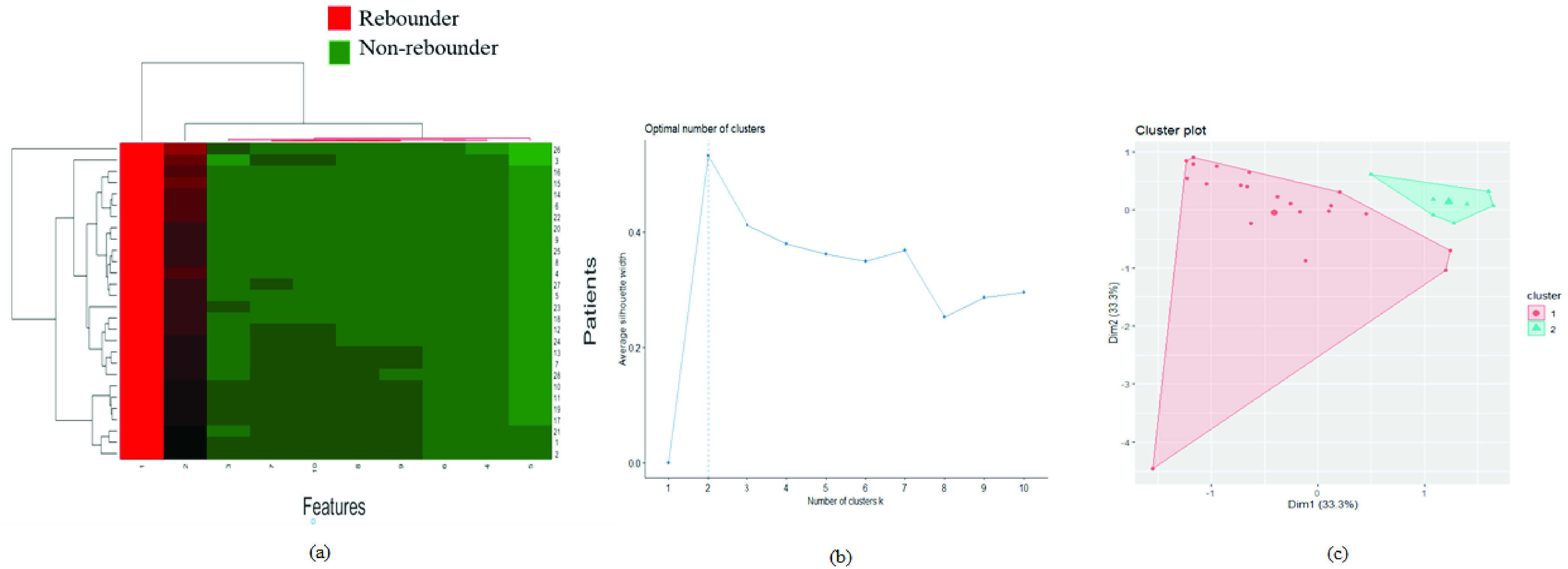
*Unsupervised clustering analysis of retinal tissue features:* (a) The clustergram of radiomics features extracted from the entire retinal tissue compartment (ILM to RPE). The X-axis represents the reduced-dimension features (10 features selected using PCA) and the Y-axis represents the number of patients. (b) The elbow curve represents an optimum number of clusters formed using the top two principal components after performing PCA on the entire retinal tissue feature set for K-mean clustering analysis. The optimum number of clusters (k) were observed to be two. (c) Clusters after performing clustering using k = 2. Rebounders and non-rebounders are included in clusters 1 and 2, respectively.

The dimension of the entire $F_{RTC}$ features were reduced using PCA and the top 10 features were selected for unsupervised hierarchical clustering (Fig. 6(a)). The red clusters included 54 percent of the rebounders and the green clusters included 82 percent of the non-rebounders.

For K-means clustering, the optimum number of classes obtained from the elbow curve (Fig. 6(b)) was two. K-means clustering was done using the top two principal components to classify the retinal tissue features into the rebounder and the non-rebounder classes. Fifty percent of the rebounders and 63 percent of the non-rebounders were included in clusters 1 and 2, respectively.

### D. Experiment 3: Combination of Fluid and Retinal Tissue Features Predict Response to Anti-VEGF Therapy

#### 1) Supervised Classification

In our third experiment, a fusion of $F_{f}$ and $F_{RTC}$ were used to assess their efficacy in distinguishing the two groups of patients. The ACC value achieved by the LDA classifier was 0.79 ± 0.06. The AUC, Sensitivity, and Specificity values were 0.78±0.08, 0.77±0.04, and 0.78 ± 0.06. The outcomes of the other classifiers are reported in Table 3.

**TABLE 3:** Classification Results of Different Classifiers on $F_{OCT}$

Classifier	AUC	ACC	Specificity	Sensitivity
LDA	0.78±0.08	0.79±0.06	0.78±0.06	0.77±0.04
QDA	0.65±0.09	0.70±0.07	0.74±0.16	0.65±0.19
RF	0.69±0.09	0.72±0.08	0.73±0.20	0.70±0.19
SVM (Gaussian Radial basis Kernel)	0.62±0.02	0.69±0.04	0.70±0.07	0.69±0.02

**FIGURE 7.**
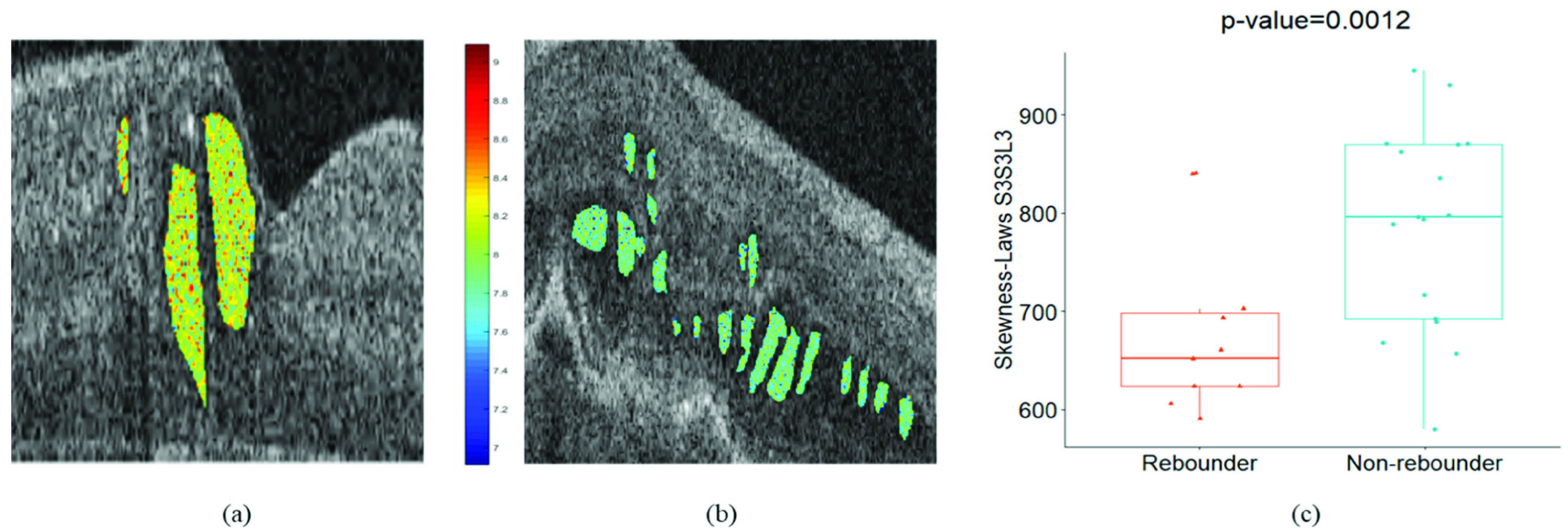
*Feature analysis for fluid and retinal tissue compartments:* (a), (b) Feature map of laws S3S3L3 feature for one case of rebounder and non-rebounder, respectively. (c). The left and the right box and whisker plot represent the feature values of Skewness-Laws S3S3L3 for the rebounders (N = 11) and non-rebounders (N = 17), respectively.

The top-performing feature selected during classification from the $\mathrm {F}_{\mathrm {OCT}}$ feature pool was the Skewness-Laws S3S3L3 IRF feature (p-value = 0.0012). The S3S3L3 feature represents the Laws energy-based textural patterns of spots (or S) in horizontal and vertical direction and levels (or L) in diagonal direction using a $3\times 3 \times 3$ convolution filter. The selection of the topmost feature from the IRF subcompartment suggests that the IRF features are able to distinguish treatment response between the rebounders and the non-rebounders. The feature map of the Laws S3S3L3 feature, as presented in Fig. 7(a) and 7(b) shows significant discrepancies in texture between the rebounders and the non-rebounders, with the rebounder having higher feature expression.

#### 2) Unsupervised Clustering Analysis

**FIGURE 8.**
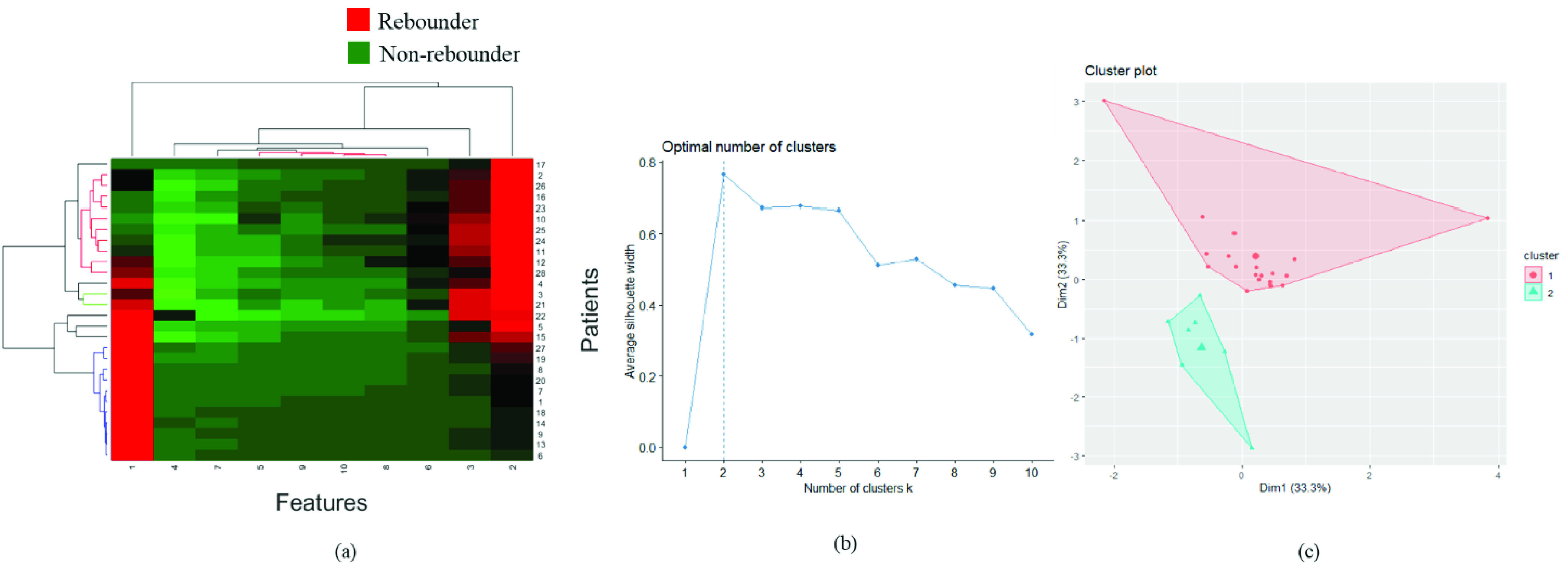
*Unsupervised clustering analysis on fluid and retinal tissue feature combination:* (a) The clustergram of the combination of fluid and retinal tissue features. The X-axis represents the reduced-dimension features (10 features selected using PCA) and the Y-axis represents the number of patients. (b) The Elbow curve represents an optimum number of clusters formed using the top two principal components after performing PCA on the entire retinal tissue feature set for K-mean clustering analysis. The optimum number of clusters (k) were observed to be two. (c) Clusters after performing clustering using k = 2. Rebounders and non-rebounders are included in cluster 2 and 1, respectively.

The hierarchical clustering performed on the top 10 features from the $F_{OCT}$ feature pool produced two clusters: the red cluster included 66 percent of the rebounders and the green cluster that included 64 percent of the non-rebounders (Fig. 8 (a)). K-means clustering, with an optimum number of clusters = 2, obtained from the elbow curve, included 64 percent rebounders within cluster 2 and 52 percent non-rebounders within cluster 1.

### E. Visual Class Activation Map Results

**FIGURE 9.**
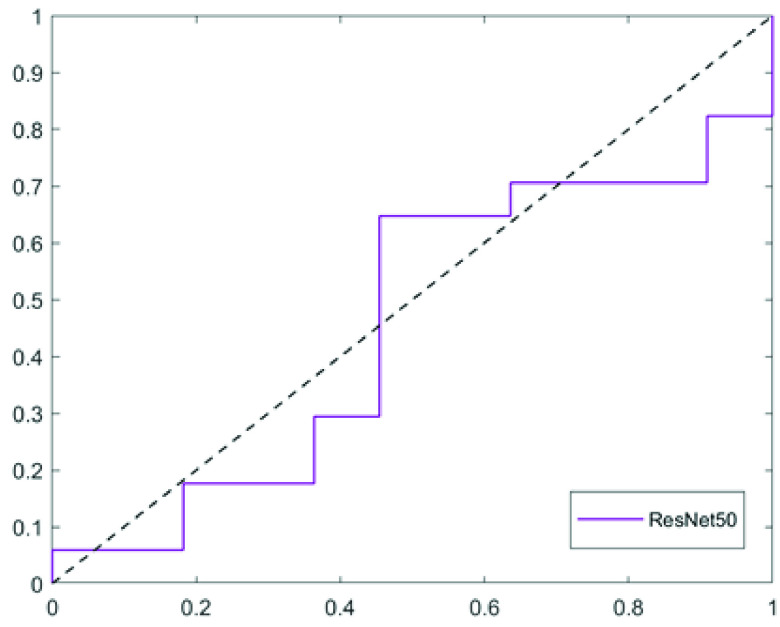
Receiver operating chracteristics curve for ResNet50.

**FIGURE 10.**
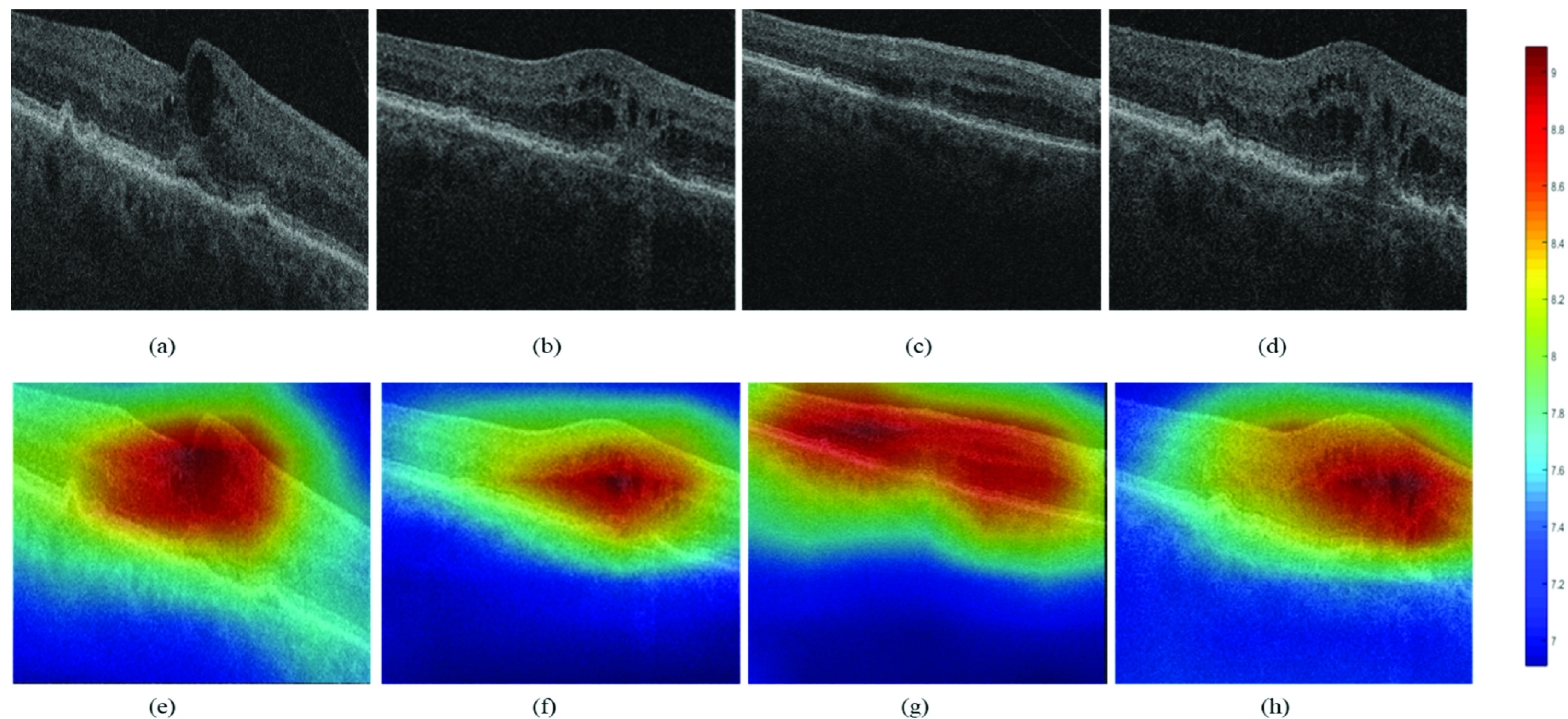
*Deep learning based class activation map (CAM) for visualization of the most discriminating region of interest corroborating the region identified by supervised machine learning on the PERMEATE study*. The train to test ratio of images was 80:20. The CAM was generated in Keras using the Gradient-weighted class activation mapping (Grad-CAM) technique. Original OCT scans for (a), (b) Rebounders and (c), (d) Non-rebounders. (e), (f), (g), (h) Corresponding CAMs generated by the ReLU modifier overlaid on the OCT scans. The gradients of the last layer are shown with respect to the previous convolutional layer.

The Visual Class Activation Map as discussed in Section III E) was generated using the Grad-CAM technique in Keras. The performance of the ResNet-50 model in classifying the rebounders and non-rebounders is presented in Table 4 and the corresponding ROC curve is shown in Fig. 9. The visual CAMs are presented in Fig. 10 for two cases of rebounders (Fig. 10(a), 10(b)) and two cases of non-rebounders (Fig. 10(c), 10(d)) with the corresponding CAMs generated by the ReLU modifier overlaid on the OCT scans presented in Fig. 10(e)–10(h). The regions highlighted in red represent the high attention areas with strong predictive power. It may be observed from Fig. 10(e)–10(h) that the attention area is localized between ILM to EZ, more specifically around the IRF subcompartment which corroborates with the region with strong discrimination ability as obtained through radiomics analysis.

**TABLE 4:** DL-Based Classification Result on PERMEATE Dataset

Model	AUC	ACC	Sensitivity	Specificity
ResNet-50	0.53±0.05	0.58±0.08	0.83±0.05	0.30±0.08

### F. Clinical Implication Assessment

In addition to discriminating between eyes that need more frequent dosing compared to those eyes that may tolerate extended dosing intervals, the radiomic features must be capable of identifying a specific group of patients with high accuracy (>90% rebounders or >90% non-rebounders). This is important in order to be able to identify those patients who might need to maintain higher frequency dosing or extended slower dosing. One way of doing so is through the balance between sensitivity and specificity corresponding to different threshold values, as illustrated in Table 5.

**TABLE 5:** Sensitivity and Specificity Values for Different Thresholds on Classification Prediction Score

Threshold	Sensitivity (%)	Specificity (%)
0.46	88.24	54.55
0.73	94.12	27.27

At a threshold of 0.46 on the classification prediction score, we could identify 88.24 percent of the non-rebounders (54.55 percent of the rebounders) correctly. As the threshold value is increased, the percentage of the correctly classified non-rebounder group is also increased to the detriment of the percentage of the rebounder group. This approach provides a potential way to gate therapeutic decision-making in a safe, but nonetheless personalized way. This type of approach could also be potentially utilized in clinical trial enrichment based on patient selection.

## V. Discussion

The main objective of this work was to evaluate (i) the role of the texture-based radiomics features to capture the morphological characteristics of the anatomic subcompartments visualized with SD-OCT to predict response to anti-VEGF therapy and (ii) the relative importance of the radiomic features extracted from the different fluid [e.g., Intraretinal Fluid (IRF) and Subretinal Fluid (SRF)] and the retinal tissue compartments [e.g., Inner Limiting Membrane (ILM) to Retinal Pigment Epithelium (RPE), Ellipsoid Zone (EZ) to RPE, ILM to EZ] for therapeutic response assessment. The rationale behind looking for the OCT-derived texture features for predicting anti-VEGF treatment response was that the unique and distinct morphological characteristics of the rebounders and the non-rebounders on their respective OCT scans can be captured and quantified by the texture-based radiomics descriptors. To address this, we performed a preliminary study to evaluate multi-compartment spatially derived radiomic features on the baseline OCT images of the PERMEATE clinical trial to predict the degree of response to anti-VEGF therapy.

In this study, we performed three experiments to evaluate our hypotheses with respect to the role of radiomic texture features and the individual spatial compartments on the OCT scans. In our first experiment, we found that the significant textural differences that exist within the retinal fluid subcompartments (IRF and SRF) between the rebounders and the non-rebounders are well captured by the texture-based radiomics descriptors. We identified that the IRF subcompartment is the most important fluid subcompartment that contains the distinct features that favorably distinguished between rebounders and non-rebounders. On evaluating the features, we observed that the Laws energy features were over-expressed for the rebounders.

The goal of the second experiment was to capture the textural discrepancies within the retinal tissue compartments (e.g., ILM-EZ, ILM-RPE, EZ-RPE) that exists among the rebounders and the non-rebounders. The retinal tissue compartment between ILM and EZ was found to contain the most discriminative features that successfully distinguished the two treatment response groups with the Laws energy descriptors differentially expressing in the rebounders.

Finally, our third experiment aimed to evaluate the overall specific subcompartments that are most relevant for the purpose of predicting response to therapy. We observed that the IRF subcompartment appeared to be the most important spatial compartment, and the features pertaining to this subcompartment were mostly associated with anti-VEGF treatment response. Higher expression of Laws energy descriptors was found for the rebounders. The experimental results suggest the potential of the texture-based radiomics descriptors in discriminating patients based on therapeutic treatment response. Laws texture-based features may be used to develop imaging biomarkers in future that could help the clinicians in therapeutic decision making and more personalized treatment planning.

Furthermore, the visual class activation maps (CAMs) obtained by the convolutional neural network (CNN) highlighted the region between ILM to EZ, explicitly the region around the IRF subcompartment, to be the most important region of interest. This finding implicitly corroborates the findings of the supervised machine classifier in Experiment 3. For practical implementation, we also presented a threshold predictor where by increasing the threshold we could identify a particular patient group that can be treated with high confidence.

Although these preliminary results are promising, we acknowledge that the study did have a number of limitations that should be discussed. The major limitation lies with the small sample size (N = 28). The complexity of the clinical question that was addressed in the present study (i.e., tolerance of treatment extension after six months of initial therapy) may not be as amenable to isolated DL methods. This therefore potentially accounts for the lower AUC and ACC values yielded by the DL models on PERMEATE study compared to the state-of-the-art methods in the literature. In recent work from our group [44], we showed that in the context of problems with small sample sizes, DL models tend to perform marginally better than random guessing. This may result in the lack of a consistent identification of regions in the visual CAMs that was learned by the DL model. A larger and homogeneous dataset may provide more consistent and robust features that can emphasize our findings. Also, a separate independent validation dataset needs to be considered to validate the experiment outcomes. Based on these two limitations, we are currently planning to evaluate these predictors in a much larger dataset, the VISTA phase III clinical trial. Additionally, in this study, we only considered OCT imaging. The other imaging modalities such as UWFA fundus images were not considered in this study but is clearly a venue for future work. Incorporation of anatomical and morphological features from other imaging modalities with radiomics may provide a better assessment of the disease prognosis. Furthermore, the compartmental analysis and evaluation of the radiomics features in the present study depend on the accurate segmentation of fluid and retinal tissue subcompartments. Although these images underwent extensive quality controls for segmentation (automated initial segmentation with sequential expert confirmation) subtle variations in segmentation are likely to occur particularly in scans of lower quality or very high pathology. This may need to be evaluated in a more rigorous future study.

## VI. Conclusion

The present study evaluates the relevance of radiomics features extracted from different spatial compartments of the retina on OCT scans to identify those patients who tolerate treatment interval extension with anti-VEGF treatment. The major findings of the present study are that texture based radiomics features are most associated with response to anti-VEGF therapy and that the texture features pertaining to the IRF subcompartment are most implicated in discriminating the rebounders from the non-rebounders, and therefore have the greatest discriminative power in predicting response to anti-VEGF treatment. These results have major implications for potential personalized decision-making in therapeutic selection and optimized identification of eyes that might benefit from combination therapeutics or emerging therapeutic strategies.
